# 3D multiscale-imaging of processing-induced defects formed during sintering of hierarchical powder packings

**DOI:** 10.1038/s41598-019-48127-y

**Published:** 2019-08-12

**Authors:** Gaku Okuma, Shuhei Watanabe, Kan Shinobe, Norimasa Nishiyama, Akihisa Takeuchi, Kentaro Uesugi, Satoshi Tanaka, Fumihiro Wakai

**Affiliations:** 1 Laboratory for Materials and Structures Laboratory, Institute of Innovative Research, Tokyo Institute of Technology, R3-23 4259 Nagatsuta, Midori, Yokohama 226-8503 Japan; 2 Japan Synchrotron Radiation Research Institute, JASRI/SPring-8, Kouto 1-1-1, Sayo, Hyogo 679-5198 Japan; 3 Department of Materials Science and Technology, Nagaoka University of Technology, 1603-1, Kamitomioka, Nagaoka, Niigata 94-2188 Japan

**Keywords:** Imaging techniques, Ceramics

## Abstract

The characterization of the processing-induced defects is an essential step for developing defect-free processing, which is important to the assurance of structural reliability of brittle ceramics. The multiscale X-ray computed tomography, consisting of micro-CT as a wide-field and low-resolution system and nano-CT as a narrow-field and high-resolution system, is suitable for observing crack-like defects with small length and with very small crack opening displacement. Here we applied this powerful imaging tool in order to reveal the complicated three-dimensional morphology of defects evolved during sintering of alumina. The hierarchical packing structure of granules was the origin of several types of strength-limiting defects, which could not be eliminated due to the differential sintering of heterogeneous microstructures. This imaging technique of internal defects provides a link between the processing and the fracture strength for the development of structural materials.

## Introduction

Sintering is an industrial process of consolidating powder compacts by heat in order to manufacture ceramic components for applications in electronics, optics, and energy systems. The production of engineering ceramics requires processing techniques, which ensure reliability and formation of complex shapes. The mechanical failure of ceramics often occurs from internal defects originally generated during powder processing such as dry pressing (uniaxial pressing, cold isostatic pressing), slip casting, pressure filtration^1^, and gelcasting^2^. The inhomogeneous density distribution of powder compacts leads to nonuniform shrinkage in sintering, and creates transient internal stress^3^. This stress influences the evolution of microstructural defects from the inhomogeneous regions of particle packing, such as agglomerates^4^. The strength of sintered body depends on the powder processing method^5^. The characterization of the processing-induced defects is the basis for developing defect-free processing, which is important to the assurance of structural reliability of brittle ceramics^6^.

X-ray computed tomography (CT) is a powerful technique to observe the microstructural evolution during sintering in the length scale from micrometer to nanometer^7–14^. Recently, Takeuchi and co-workers at SPring-8 synchrotron facility developed a multiscale X-ray computed tomography consisting of an X-ray microtomography as a wide-field and low-resolution system and a phase-contrast high-energy X-ray nanotomography as a narrow-field and high-resolution system^15^. This multiscale-CT measurement is suitable for observing crack-like defects with small length (a few tens of micrometers) and with very small crack opening displacement (COD). The distribution of defects in the entire body of a sample is examined with micro-CT to identify the position of the internal defect. The shape of the identified defect is observed nondestructively by nano-CT. We demonstrate here that this technique reveals the evolution of process-induced defects in sintering of hierarchical powder packings.

Dry pressing, or powder compaction, is a common process to obtain powder compacts. Hierarchical powder packing structure is formed by this process, because large granules consisting of fine primary particles are used in order to improve the flowability of the powder during die filling. The morphologies of granules, which are prepared by spray-drying, are either “solid” or “hollow”^16^. When the wall of a hollow granule collapses, a dimple or a crater is formed on the surface. As dimples and spherical cavities of hollow granules remain after the powder compaction process^17,18^, they develop into circular crack-like defects after sintering^19^. The crack-like defects are also formed along boundaries of granules^20^. Although a conventional X-ray CT was used to observe coarse pores in sintered alumina, the crack-like defects were not detected clearly because COD was smaller than the spatial resolution^21,22^.

The aim of the present work is to reveal the complicated evolution process of three-dimensional morphology of defects during sintering by using the multiscale-CT. This technique provides the data necessary for predicting the mechanical reliability of the products, i.e., size, shape, orientation, and distribution of strength-limiting defects. This powerful imaging tool will open new perspectives for developing defect-free powder processing and sintering.

## Results

### Types of processing-induced defects

**Figure 1:**
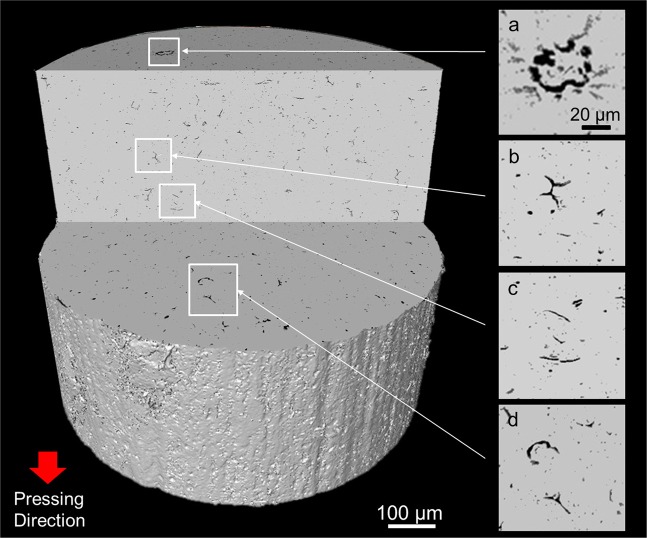
Micro-CT image of defects on cross-sections of sintered alumina (relative density, *ρ* = 98%). The axial direction is the direction of uniaxial pressing of the powder.

**Figure 2:**
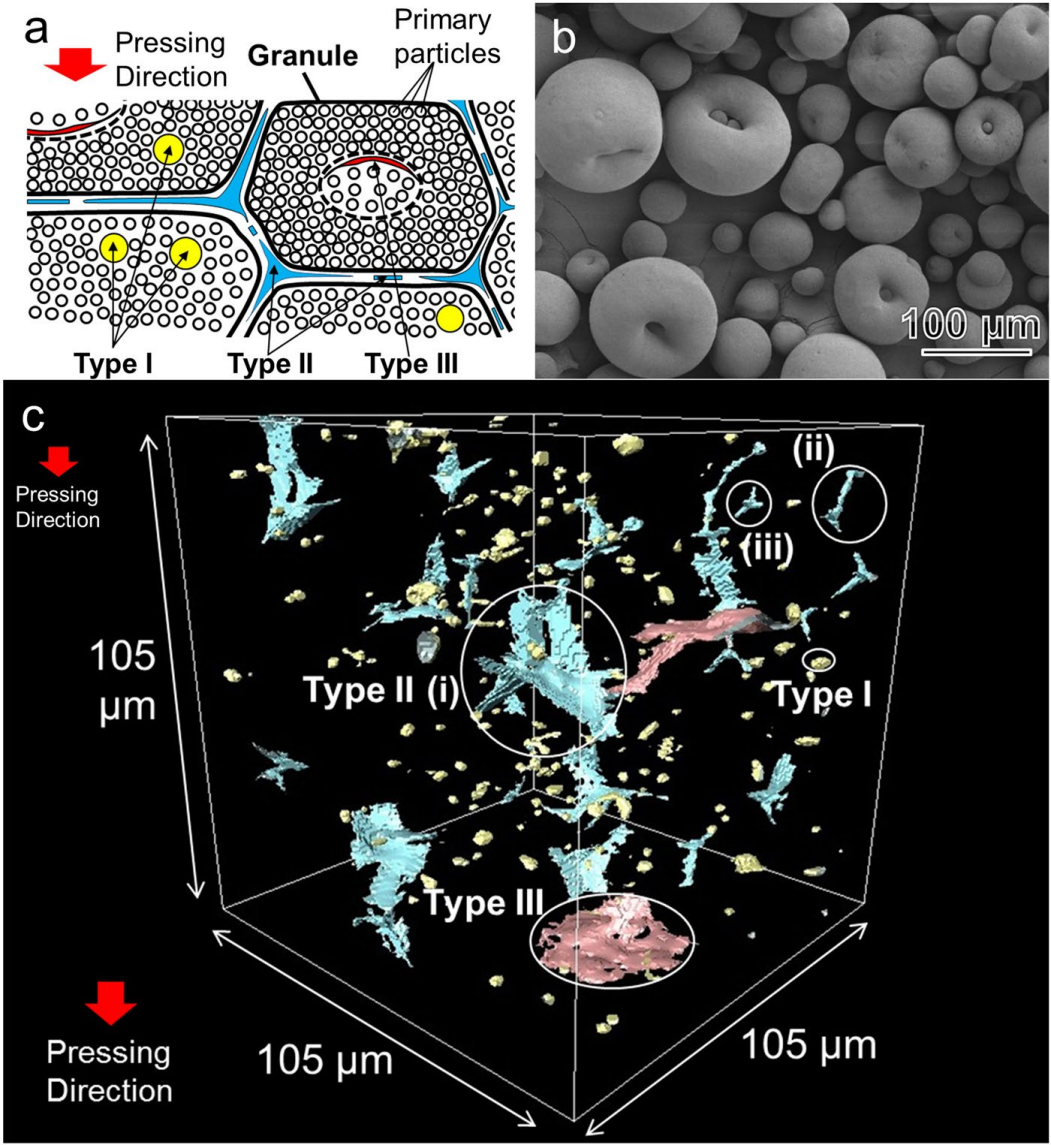
Types of processing-induced defects. (**a**) Relation between defects and hierarchical powder packing structure. Type I defects arise from intragranule porosity. Type II defects are formed at boundaries among granules. Type III defects are formed from the cavity of hollow granules. (**b**) SEM image of alumina granules. There are large dimples in large granules, while dimple is small or apparently absent in small granules. (**c**) 3D micro-CT images of defects in sintered alumina (*ρ* = 98%). Coarse round-shaped pores (Type I), branched crack-like defects (Type II), and circular crack-like defects (Type III).

Figure 1 shows micro-CT image of a cylindrical specimen of sintered alumina (Al_2_O_3_, relative density *ρ* = 98%, measured by the Archimedes method). The axial direction is the direction of uniaxial pressing of the powder. Any cross-section can be observed non-destructively. Numerous coarse round pores, and some large branched crack-like defects (b) are scattered in the cross-section. Furthermore, semicircular crack-like defects (a, d) are observed in the cross-section perpendicular to the axis, while linear crack-like defects (c) are seen in the cross-section parallel to the axis. The observed defects are related to the hierarchical powder packing structure (Fig. 2a), which is formed by packing of granules containing dimples (Fig. 2b). The three-dimensional (3D) structures of defects are displayed in Fig. 2c (see also Supplementary Movie 1). There are so many defects of different shapes in the dense alumina ceramics with the residual porosity of 2%. But, the total volume of defects observed by micro-CT was only about 1%, one half of the residual porosity, because the width of the crack-like defect is very thin, and fine pores smaller than the voxel size of 0.5 μm could not be imaged. The defects are classified into three types; coarse round pores with diameters about 10 μm (Type I), branched crack-like defects (Type II), and circular crack-like defects (Type III), the crack plane of which oriented vertically to the direction of uniaxial pressing.

**Figure 3:**
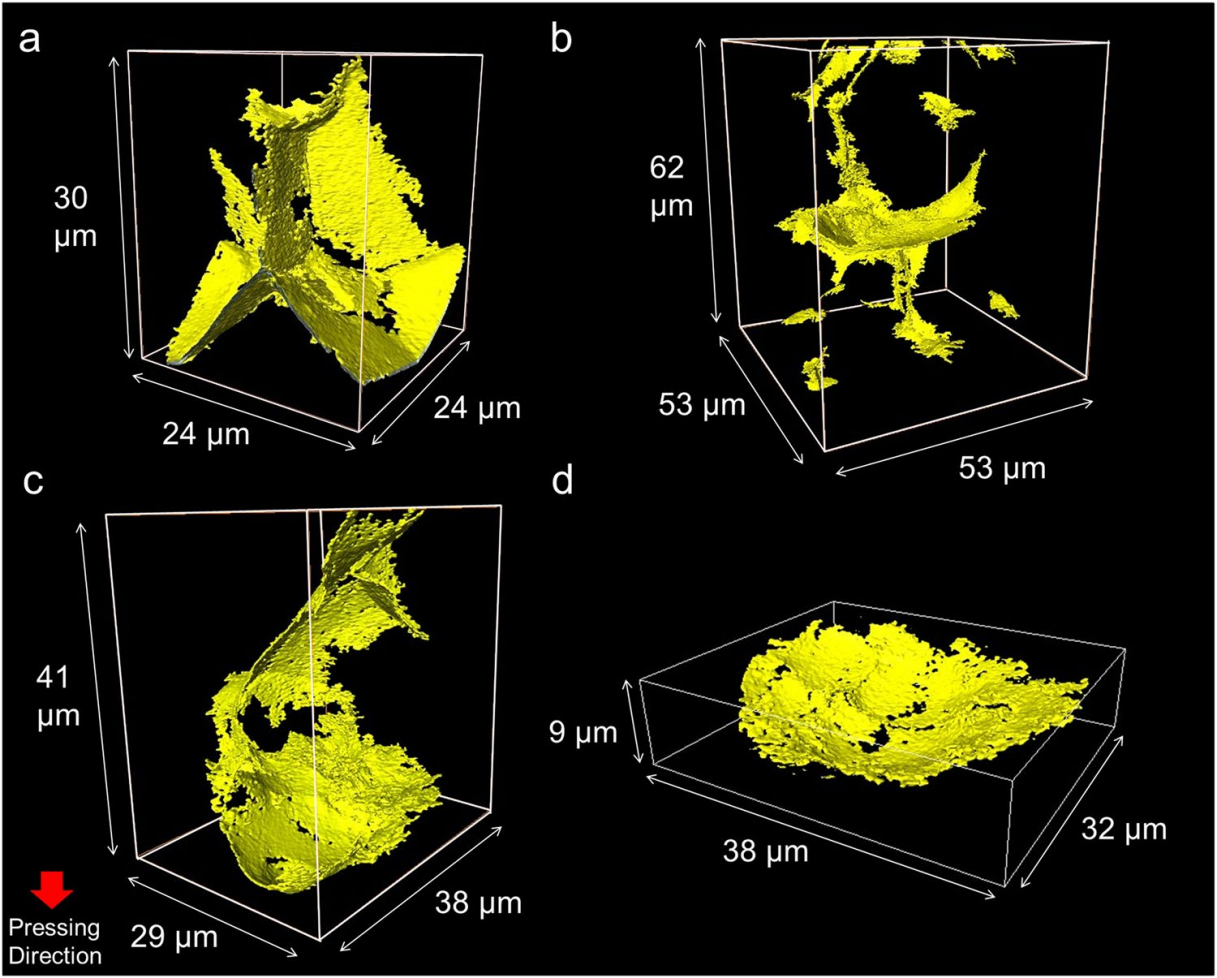
Nano-CT images of defects. (**a**–**c**) Branched crack-like defects (Type II), (**d**) shallow bowl-shaped crack (Type III).

There are numerous variations in the shape of type II defects. For example, the type II defect in Fig. 2c (i) was observed by nano-CT as shown in Fig. 3a and Supplementary Movie 2. The nano-CT image clearly indicates that the type II defects are associated with cell structures formed by the compaction of granules (Fig. 2a). The branched crack-like defects consist of faces, which are planar boundary between granules. Three faces meet at a linear triple junction, which, in turn, meets at a quadruple point. The micro-CT image in Fig. 2c illustrates also (ii) the linear defects along the triple junction, and (iii) four linear defects meeting at a quadruple point. Figure 3b and Supplementary Movie 3 present a case where a planar defect, which is perpendicular to the direction of uniaxial pressing, is connected with several linear defects along the triple junctions. Figure 3c and Supplementary Movie 4 display that a defect surrounding a granule is connected to other defects surrounding other granules. The total length of connected type II defects can be larger than the size of a single granule.

The detailed shape of a type III defect is observed by nano-CT mode in Fig. 3d. The shallow bowl-shaped crack is seen as a semicircular crack in the cross-section perpendicular to the axis in Fig. 1a,d, while it is depicted as a slightly curved linear crack in the cross-section parallel to the axis (Fig. 1c). Supplementary Figures S1, S2 and Supplementary Movie 5 describe the cross-sectional observation of the shallow bowl-shaped crack in more detail. The circular type III defect is distinguished from planar type II defects (Fig. 3b), which are also perpendicular to the axis, but, are connected with several linear defects along triple junctions.

### Evolution of defects during sintering

**Figure 4:**
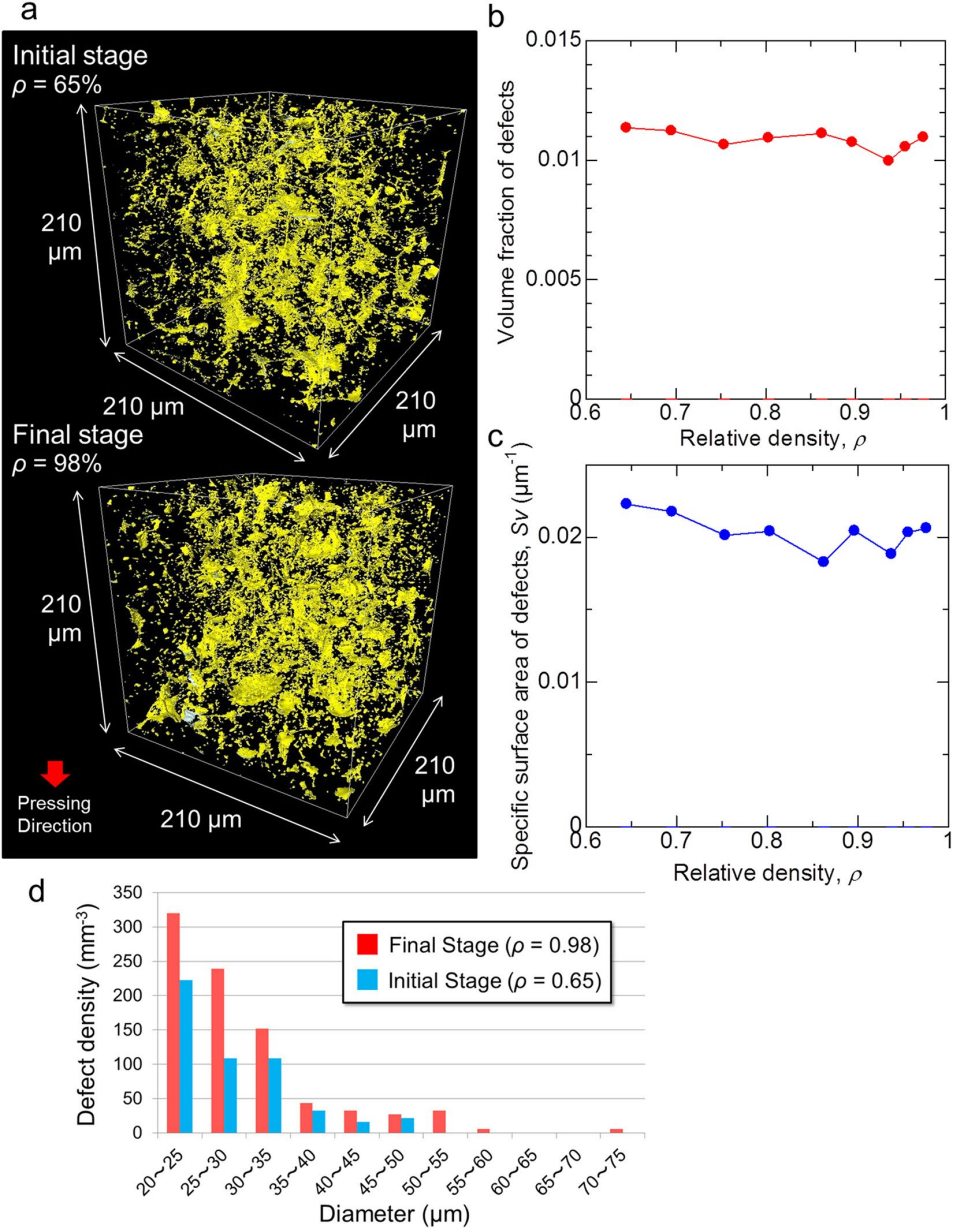
Evolution of defects during sintering. (**a**) 3D view of defects observed by micro-CT at the initial stage (*ρ* = 65%) and the final stage (*ρ* = 98%). (**b**) Volume fraction of defects. (**c**) Specific surface area of defects. (**d**) Size distribution of circular crack-like defects (Type III) in a volume element of 420 × 420 × 1050 µm^3^.

All three types of defects were observed by micro-CT not only in the final stage (*ρ* = 98%), but also in the initial stage (*ρ* = 65%) of sintering as presented in Fig. 4a. The defects had been already formed in the initial stage just after the burning-out of binder, which segregated at boundaries of granules^23^. The volume fraction and the specific surface area of defects observed by micro-CT are plotted as a function of relative density in Fig. 4b,c, respectively. They were almost constant during sintering process. This means that the densification occurs by the shrinkage of fine pores (less than 0.5 µm) among primary particles, which cannot be observed by micro-CT.

The statistical analysis was conducted to evaluate the evolution of circular crack-like defects (Type III). Figure 4d shows the distribution of diameter of type III defects (also see supplementary Tables 1, 2). The diameter and number of large type III defects in the final stage increased from those in the initial stage. The average COD of top 10 largest type III defects in the initial and final stages were 1.4 µm and 1.8 µm, respectively (See Supplementary Fig. S8). Thus, the large crack-like defects did not shrink, but grew slightly during sintering. It is difficult to eliminate the processing-induced defects by sintering, so that they remain as residual defects. The increase in the number of type I pores occurred also when the diameter was larger than 5 µm, but, the number of pores smaller than that decreased with densification (see Supplementary Fig. S3, Supplementary Table 3). The conditions for the defect growth will be discussed later.

**Figure 5:**
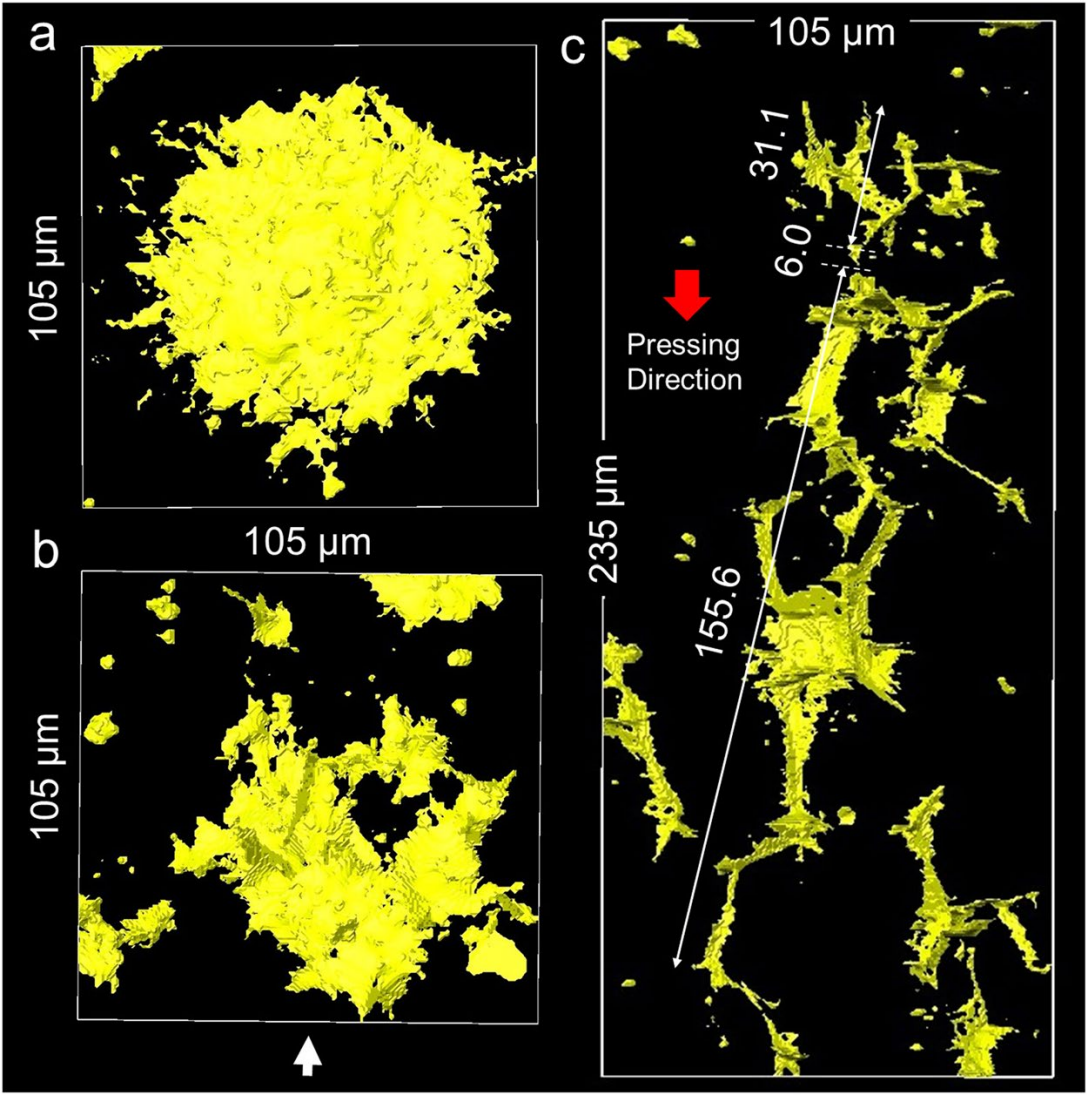
Large strength-limiting defects. (**a**) Top view of the largest circular crack-like defect (Type III). (**b**) Top view of the network of branched crack-like defects (Type II). (**c**) Side view of the network of branched crack-like defects in the direction of white arrow.

The micro-CT is an efficient method to observe the distribution of large strength-limiting defects. In order to find the potential fracture origin, largest defect was searched among nine samples of different relative density (64–98%). The total volume for the search was ${V}_{E}^{micro}$ = 1.57 mm^3^. The largest defect was a circular crack-like defect (Type III) with a diameter of 87.5 µm (Fig. 5a), which was found in a sample with a relative density of 95.6%. On the other hand, the total length of the network of branched crack-like defects (Type II) was more than 100 µm as highlighted in Fig. 5b,c, which was also found in a sample with relative density of 95.6%. The maximum diameter of round pores (Type I) was 18 µm in the final stage (98%).

## Discussion

### Origin of processing-induced defects

The present results of multiscale-CT provide a unified view on the origins of defects as schematically illustrated in Fig. 2a. The coarse round pores (Type I) distributed randomly, then, they arise from intragranule porosity, which has been observed by Cottrino using X-ray tomography^18^. The branched crack-like defects (Type II) are formed at boundaries among granules. Shinohara classified type I and type II defects in sintered alumina by using scanning electron microscopy (SEM)^20^. The circular crack-like defects (Type III) are formed from the cavity or the dimple of hollow granules. Sato found such crack-like flaws by SEM observation, and identified that they appeared as circular dark areas inside granules by optical microscopy in transmission mode^19^. Indeed, some large type III defects were surrounded by type II defects in the initial (Supplementary Fig. S4a and Supplementary Movie 6) and the final stage of sintering (Supplementary Fig. S4b and Supplementary Movie 7). The formation of defects at boundaries among granules depends on the shape and size of granules^24^, the properties and distribution of binders^25,26^, the binder content^22^, the moisture content which affects the glass transition temperature of binder^27–29^, the compaction behavior, and the binder burnout process.

Large voids and cracks in a uniform porous matrix shrink as the matrix densifies in free sintering where no constraint is imposed. Flinn demonstrated that the diameter of spherical pore decreased linearly with the macroscopic shrinkage^30^, in contrast to the classical supposition^31^. The large pore can shrink because the thermodynamic driving force increases with decreasing pore coordination number, which occurs by particle rearrangement^32^ and grain growth^33^. Martin simulated the closure of a crack in free sintering by discrete element method (DEM)^34^. He also pointed out that some form of constraint was necessary for a preexisting crack to grow during sintering. The diameter of circular crack-like defects and COD increased slightly with densification in the present experiment. The shrinkage of the defect is hindered by the differential sintering in heterogeneous microstructures^3,4,35,36^. Suppose a crack-like defect with the shape of a shallow bowl lies along the boundary between the dense region and the porous region (Fig. 2a), which has been formed by the compaction of a hollow granule. When the porous region shrinks faster than the dense region, the crack-like defect will grow slightly with densification. The size distribution of type III defects in the initial stage shifts toward larger sizes in the final stage (Fig. 4(d)). In a similar way, if there is a difference in shrinkage rate between adjacent granules, it will hinder the shrinkage of type II defect among two granules. The type II defects will remain in the sintering of granules of different shapes and sizes. Actually, Hondo observed the nonuniform relative motion of granules during sintering^21,22^, probably due to the different shrinkage rate of each granule. On the other hand, when small type I pores (<5 μm) are surrounded by relatively uniform matrix, they can shrink during sintering. Once large defects are formed in powder processing and the burn-out process, the differential sintering due to the microstructural heterogeneity hinders the elimination of these defects during normal sintering. In order to improve the reliability of sintered products, the present observation suggests that the improvements for defect-free powder processing is the most important approach^5^. Otherwise large defects must be eliminated by the application of external stress during sintering such as hot pressing, hot isostatic pressing^37^, sinter forging^38^, and spark plasma sintering.

### Fracture strength

The dense alumina with the relative density of 98% contains three types of defects, which will be principal sources of fracture in different ways. The round type I pore (cavity, or void) causes a stress concentration. When other defects locate at the immediate vicinity of a pore, the interaction between the defect and the pore produces the fracture^6^. The pore-induced failure was analyzed by Baratta^39^ and Evans^40^ assuming either a circumferential crack around a spherical pore^39,40^ or semicircular cracks in the vicinity of a spherical pore^40^. We could not detect such circumferential cracks nor semicircular cracks around the type I defects, because the voxel size was still larger than the COD of microcracks. Zimmermann estimated that the length of semicircular cracks was 4–8 times the value of average grain size^41^. For the alumina with the average grain size of 0.75 μm, the maximum pore radius (*R* = 9 μm) and the possible length of semicircular cracks (*c* = 4–6 μm, *c*/*R* = 0.44–0.67) gives the stress intensity factor equivalent to the circular crack with the diameter of 31–36 μm by applying Zimmermann’s analysis (Supplementary notes). We conclude that large type II and type III defects are the strength-limiting defects of sintered alumina fabricated by dry pressing, because their sizes are much larger than the equivalent size of type I defect. Of course, the spherical pore with microcracks is the major source of fracture in sintered alumina fabricated by slip casting a slurry^30^, where type II and type III defects are not formed.

The fracture strength of ceramics is limited by the largest pre-existing flaw size, and is proportional to the critical stress intensity factor *K*_*c*_;

1 $$\sigma =Y{K}_{C}/\sqrt{a}$$

where 2*a* is the flaw size, and *Y* is a geometry factor. With the knowledge on the size, shape and orientation of largest defects observed by micro-CT (Fig. 5), it is possible to predict which defect will be responsible for the fracture strength under a specific loading condition. The tensile strength relates primarily to the presence of internal defects, while the flexural strength often reflects the surface crack population. When a tensile stress is applied parallel to the uniaxial pressing direction, the fracture will occur from the largest type III defect with the diameter of 87.5 µm (Fig. 5a).

The fracture resistance of alumina increases with crack extension by crack-tip shielding mechanisms such as grain bridging^42^. The fracture strength at realistic flaw size is not governed by fracture toughness measured by macroscopic cracks, but the toughness at the crack tip, *K*_0_. Seidel and Rödel^43^ revealed that the crack tip toughness of alumina was 2.3 MPa·m^1/2^, while the fracture toughness measured by using macroscopic crack was 3.6 MPa·m^1/2^. Yoshida reported *K*_0_ of 2.8 MPa·m^1/2^ by using micro-cantilever beam specimens^44^. The fracture strength is estimated to be 310–380 MPa by using $Y=\sqrt{\pi }\,/\,2$ for a circular crack and *K*_0_ of 2.3–2.8 MPa·m^1/2^ for alumina. Hondo measured the average strength and Weibull modulus of alumina in four-point bending test (standard JIS R 1601:2008), and they were ${\sigma }_{avg}^{bending}$ = 332.3 MPa and *m* = 8.5, respectively^22^. The fracture strength of ceramics decreases with increasing the specimen size, because the possibility to contain large defects increases with the specimen size. The relation between the average tensile strength ${\sigma }_{avg}^{micro}$ of the region observed by micro-CT and the average strength of four-point bending test ${\sigma }_{avg}^{bending}$ is given as;

2 $${\sigma }_{avg}^{micro}/{\sigma }_{avg}^{bending}={({V}_{E}^{bending}/{V}_{E}^{micro})}^{1/m}$$

where ${V}_{E}^{micro}$ is the observed specimen volume and ${V}_{E}^{bending}$ is the effective volume which is defined from the stressed volume of the four-point bending specimen (*V* = 3 × 4 × 10 mm^3^) and the Weibull modulus (${V}_{E}^{bending}=V(m+3)/6{(m+1)}^{2}$). We searched for the largest defect in the total volume of nine specimens. From the values ${V}_{E}^{bending}$ of 2.55 mm^3^ and ${V}_{E}^{micro}$ of 1.57 mm^3^, ${\sigma }_{avg}^{micro}$ will be 352 MPa, which is in the range of fracture strength predicted from the observed type III defect size. As the type III defects are formed inside hollow granules, their size distribution depends on the distribution of granule size. The fracture strength is influenced by the size distribution of hollow granules^24^. Nakamura measured the size distribution of circular dark areas inside granules by optical microscopy in transmission mode, and assumed that it is the same with the size distribution of circular cracks^45^. The average strength and Weibull modulus predicted from the size distribution agreed well with the experimentally measured values.

However, when the tensile stress is applied vertical to the uniaxial pressing direction, the fracture will never occur from type III defects, but will occur from large type II defects in Fig. 5b,c. Although the stress intensity factor for an arbitrary three-dimensional crack may be calculated by using, for example, the extended finite element method (XFEM), there is no simple analytical method to estimate the fracture strength from the size of type II defects. While the diameter of type III defect is smaller than the granule size, the effective size of two contiguous type II defects may exceed the average granule size. The strength of two interacting cracks can be evaluated by assuming a large crack which circumscribes them^46^. Since the types of fracture origins are different, the average strength under the tensile stress vertical to the uniaxial pressing direction is not necessarily the same with that under the stress parallel to the uniaxial pressing direction. Although Shui observed the development of anisotropic microstructure in the sintering of uniaxially pressed alumina compacts^47^, as far as the authors are aware, there is no report on the anisotropy in fracture strength of normally sintered alumina. We suppose that the wide width of strength distribution makes it difficult to detect anisotropy.

In summary, we show that the synchrotron X-ray multiscale-CT is a powerful imaging tool that reveals the complicated evolution process of three-dimensional morphology of defects during sintering. Micro-CT reveals the distribution of large strength-limiting defects in the entire body of a sample and enables us to perform the statistical analysis for evaluating the evolution of defects, while the detailed shape of a complex defect can be observed by nano-CT. Our results in this study will open the way to control internal defects formed during powder processing for improving mechanical reliability of not only alumina, but also all ceramics.

## Methods

### Materials

Industrial spray-dried α-alumina granules (DS31, Taimei Chemical Co. Tokyo, Japan) were used in this study (Fig. 2b). The granule consisted of primary particles of high purity α-alumina with the average size of ∼150 nm (Supplementary Fig. S5). The granule diameters fell within the range of 10–120 µm. The granule had a relative density of 63%, and contained organic binder of 4.4 wt%^22^. Cylindrical compacts (diameter = 10 mm, height = 5 mm) were formed by uniaxial pressing at 40 MPa and cold isostatic pressing at 200 MPa. Sintering was conducted at a slow heating rate of 3 °C/min for binder burnout in air up to 1150–1300 °C with a holding time between 0 and 60 min. The relative density of sintered body ranged from 65% to 98%. Cylindrical sample with the diameter of 0.85 mm were fabricated by laser beam machining for the multiscale-CT observation.

### Multiscale-CT

The experiments were performed at BL20XU, of the Japanese synchrotron radiation facility, SPring-8^15^. Supplementary Fig. S6a,b show the schematic drawing of experimental setup for micro-CT and nano-CT, respectively. X-ray energy of 20 keV was chosen for micro- and nano-CT mode. The sample was rotated by steps of 0.1° up to 180°. Voxel sizes for micro- and nano-CT mode were 0.5 μm and 60 nm, respectively. The measuring time for one sample was only 8 minutes. The details of the synchrotron multiscale-CT and the comparison with the conventional CT are described in Supplementary notes.

### Image analysis

The 3D mappings were reconstructed from the acquired data by using the filtered back-projection method. The 3D visualization and geometrical measurements were performed using Amira (VSG, Burlington, Massachusetts, USA), and a Gaussian filtering was applied to reduce the noise in 2D images. Local thresholding method was used to segment the gray value image into defect and material, so as to determine the defect size. The surface was discretized using triangular meshing, from which the volume and surface area of defects were calculated.

## Supplementary information

Supplementary Information

Supplementary Movie 1

Supplementary Movie 2

Supplementary Movie 3

Supplementary Movie 4

Supplementary Movie 5

Supplementary Movie 6

Supplementary Movie 7

## Data Availability

No datasets were generated or analysed during the current study.
