# Supplementary material for: 3D multiscale-imaging of processing-induced defects formed during sintering of hierarchical powder packings
Source: Sci Rep. 2019 Aug 12;9:11595. doi: 10.1038/s41598-019-48127-y (PMC6690982; doi:10.1038/s41598-019-48127-y)
Supplement: Supplementary file 1 — Supplementary Information [file 41598_2019_48127_MOESM1_ESM.pdf]

**Supplementary Information**

# 3D multiscale-imaging of processing-induced defects formed during sintering of hierarchical powder packings

Gaku Okuma[1], Shuhei Watanabe[1], Kan Shinobe[1], Norimasa Nishiyama[1], Akihisa Takeuchi[2], Kentaro Uesugi[2], Satoshi Tanaka[3] & Fumihiro Wakai[1]

[1]Laboratory for Materials and Structures Laboratory, Institute of Innovative Research, Tokyo Institute of Technology, R3-23 4259 Nagatsuta, Midori, Yokohama, 226-8503, Japan

[2]Japan Synchrotron Radiation Research Institute, JASRI/SPring-8, Kouto 1-1-1, Sayo, Hyogo 679-5198, Japan

[3]Department of Materials Science and Technology, Nagaoka University of Technology, 1603-1, Kamitomioka, Nagaoka, Niigata, 94—2188, Japan

**Supplementary Note 1. Synchrotron multiscale X-ray CT by combining micro-CT and nano-CT.**

The experiment has been performed at BL20XU, of the Japanese synchrotron radiation facility, SPring-8[1]. Supplementary Figs. S6 a,b show the schematic drawing of experimental setup, and Supplementary Fig. S6 c shows the picture of the 1st hatch at BL20XU. X-ray energy of 20 keV is chosen for micro- and nano-CT mode. Optical system of nano-CT mode is based on a phase contrast X-ray full-field microscope. A hollow-cone illumination system using a condenser zone plate (CZP), sample stages, a Fresnel zone plate (FZP) objective, and a Zernike phase plate (phase ring) are placed at the experimental hutch 1 of the BL20XU. A visible-light conversion type X-ray image detector (C12849-SY69701, Hamamatsu Photonics) is installed at the 2nd hutch located ~160 m downstream from the 1st hutch. The pixel size of the detector is 9.52 μm. Another X-ray camera can be installed between the sample and the A-FZP objective, to observe projection image of sample. Therefore, micro- and nano-CT mode can be easily switched only by inserting and removing the hollow-cone illuminating system and the X-ray camera for micro-CT. The sample was rotated by steps of 0.1° up to 180°. Voxel sizes for micro- and nano-CT mode are 0.5 μm and 60 nm, respectively.

**Supplementary Note 2. Comparison between lab-scale and synchrotron X-ray micro CT.**

Hondo[2, 3] used a conventional lab-scale micro-CT (SkyScan 1172, BRUKER AXS) to observe coarse pores in sintered alumina, but, the crack-like defects were not detected because the crack opening displacement was smaller than the spatial resolution. We observed the internal structure of the same sample ($\rho$ = 98 %) by using the conventional micro-CT, and compared with the image obtained by the synchrotron micro-CT (Fig. 2 c). Supplementary Figure S7 a and b show examples of 2D slices obtained by the synchrotron micro-CT and the conventional micro-CT, respectively. While the crack-like defects are observed clearly by the synchrotron micro-CT, they cannot be observed by the conventional micro-CT. 3D visualization by conventional micro CT shows only numerous closed pores with the size < 20 μm (Supplementary Fig. S7 c).

**Supplementary Note 3. Equivalent size of type I defect**

The average stress intensity factor at a semicircular cracks emanating from a

spherical pore is given as[4]

$$K = \sigma\left(\pi c\right)^{1/2} F_c\left(c/R\right) \tag{1}$$

where $\sigma$ is the applied stress, $R$ is the radius of the pore, $c$ is the radius of the semicircular crack, and $F_c$ is a function of $c/R$. The stress intensity factor of a circular crack is

$$K = 2\sigma\left(a/\pi\right)^{1/2} \tag{2}$$

where $2a$ is the diameter of the crack. The spherical pore with semicircular crack is equivalent to the circular crack with radius $a$

$$a = \left(\pi F_c\left(c/R\right)/2\right)^2 c \tag{3}$$

**Supplementary References**


1. Takeuchi, A., Uesugi, K., Uesugi, M., Yoshinaka, F., Nakamura, T. Nondestructive multiscale X-ray tomography by combining microtomography and high-energy phase-contrast nanotomography. Microsc. Microanal. 24 (2), 106-107 (2018).
2. Hondo, T., Kato, Z., Yasuda, K., Wakai, F. & Tanaka, S. Coarse pore evolution in dry-pressed alumina ceramics during sintering. Adv. Powder Technol. 27, 1006-1012 (2016).
3. Hondo, T., Yasuda, K., Wakai, F. & Tanaka, S. Influence of binder layer of spray-dried granules on occurrence and evolution of coarse defects in alumina ceramics during sintering. J. Euro. Ceram. Soc. 38, 1846-1852 (2018).
4. Zimmermann, A. Hoffman, M., Flinn, B.D., Bordia, R.K., Chuang, T-J., Fuller Jr., E.R. & Rödel, J. Fracture of alumina with controlled pores. J. Am. Ceram. Soc. 81, 2449-2457 (1998).

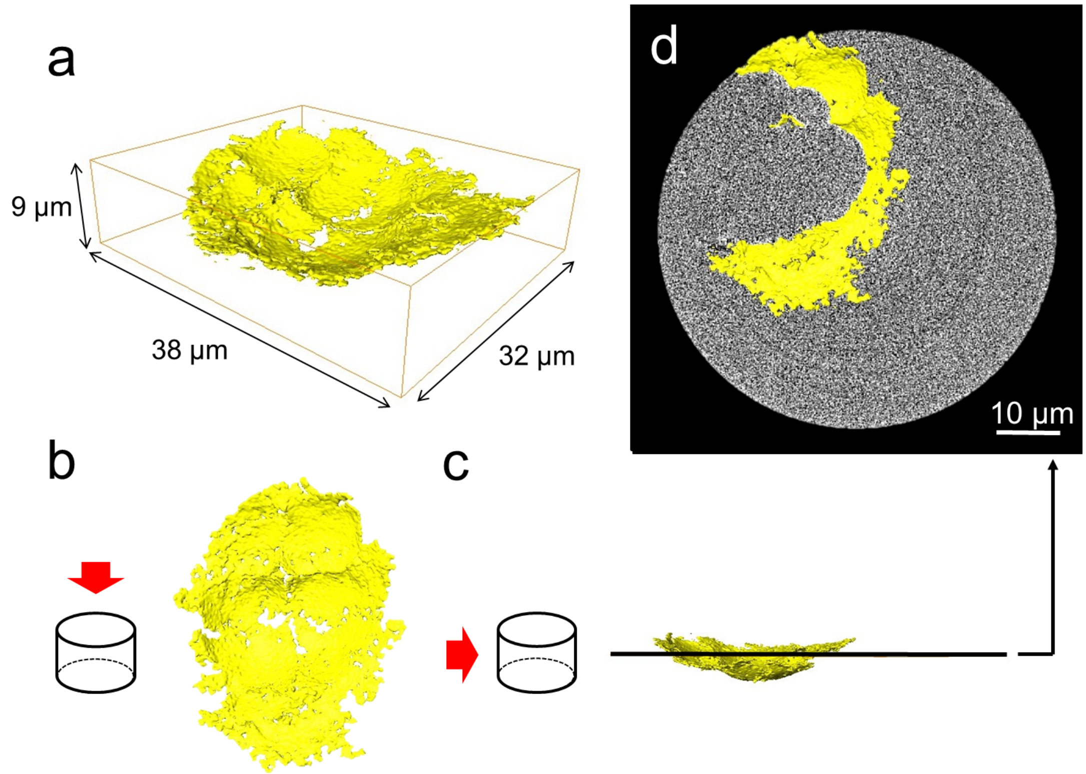


**Supplementary Figure S1. Nano-CT image of a shallow bowl-shaped crack (Type III) at the initial stage of sintering ($\rho$ = 68 %).** (**a**) 3D view. (**b**) Top view. (**c**) Side view. (**d**) Cross sectional image at the black line shown in (c).

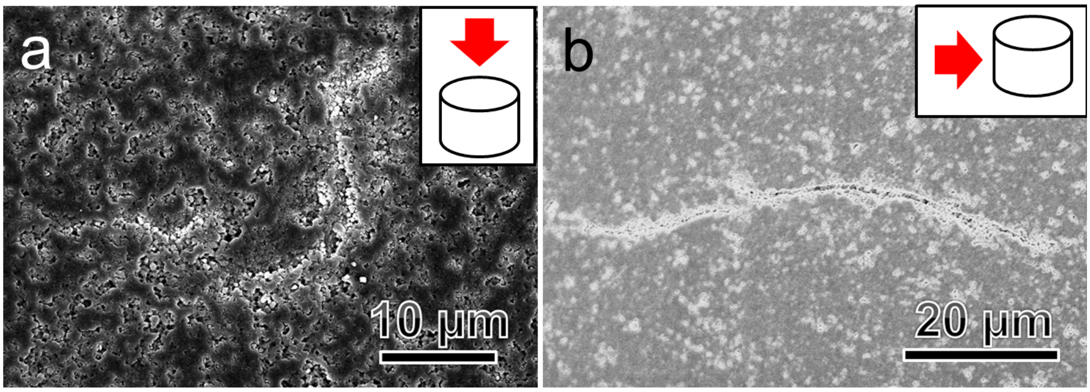


**Supplementary Figure S2. SEM images of a shallow bowl-shaped crack (Type III) ($\rho$ = 98%).** (**a**) The direction perpendicular to the pressing direction. (**b**) The direction parallel to the uniaxial pressing direction.

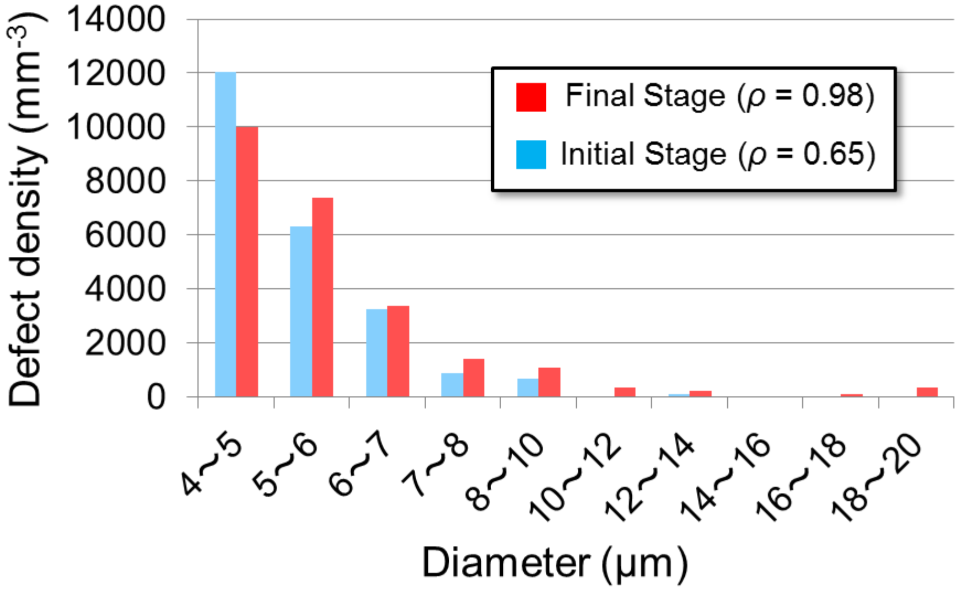


**Supplementary Figure S3. The statistical analysis of the number and size of coarse round pore (Type I).** Observed volume is 210×210×210 μm$^3$ and total about 230 defects are measured.

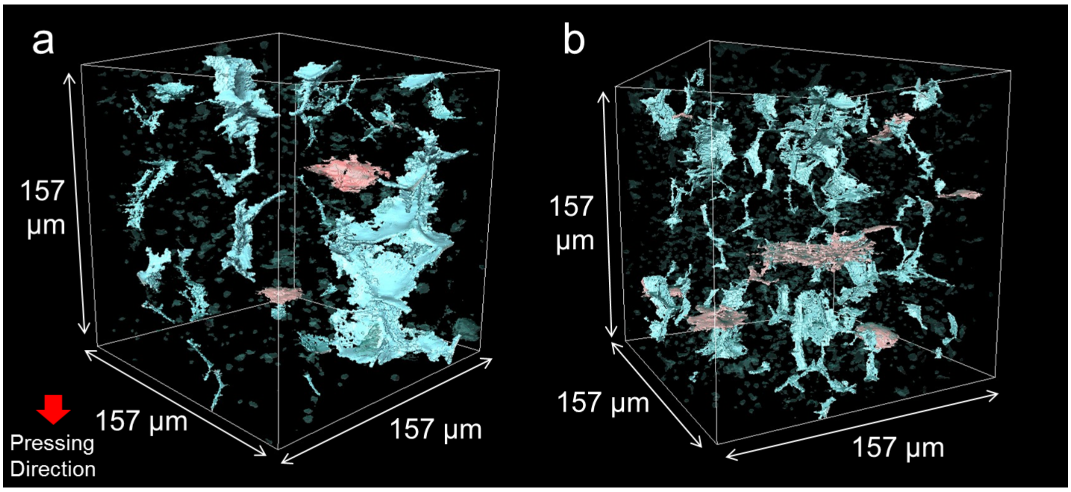


**Supplementary Figure S4. Micro-CT image of type III defects surrounded by Type II defects.** (**a**) Initial stage ($\rho$ = 66%). (**b**) Final stage of sintering ($\rho$ = 98%). Type I defects are shown as transparent.

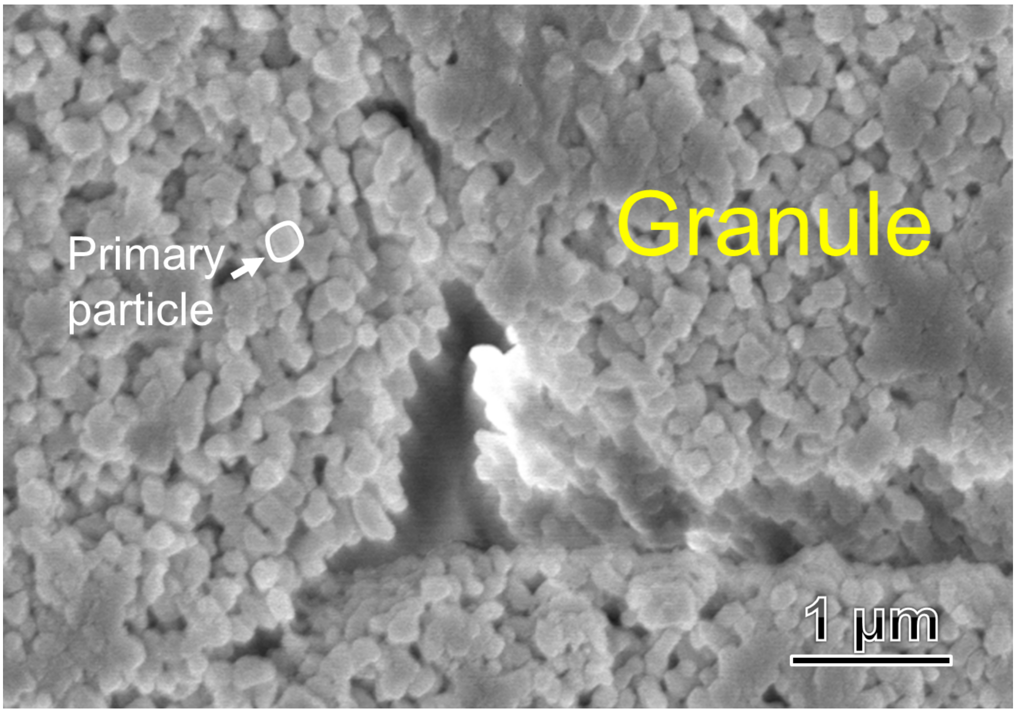


**Supplementary Figure S5. SEM image of primary particles.** A granule consists of primary particles with submicron size.

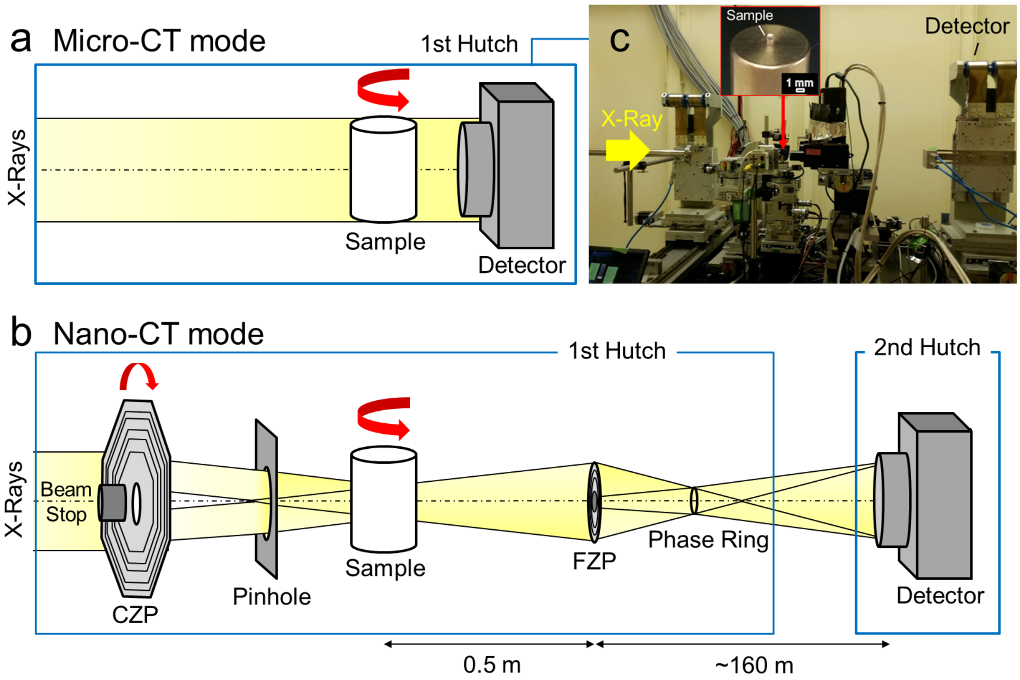


**Supplementary Figure S6.** Experimental setup of the multiscale-CT at BL20XU of SPring-8. Schematic image of (**a**) micro-CT mode, (**b**) nano-CT mode. (**c**) Real picture of 1st experimental Hutch. Two measurement modes of the micro-CT and nano-CT can be easily switched only by inserting and removing the hollow-cone illuminating system and the X-ray camera for micro-CT.

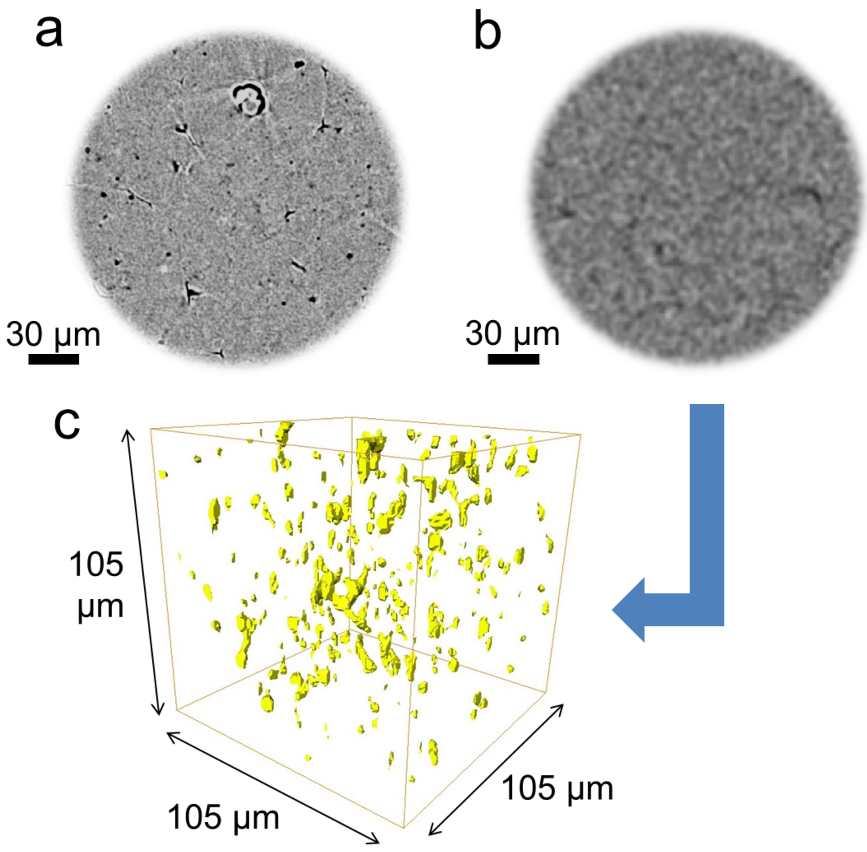


**Supplementary Figure S7.** Micro-CT images of an alumina sample with the relative density of 98%. (**a**) Synchrotron (BL20XU of SPring-8) and (**b**), (**c**) Lab-scale (SkyScan 1172) X-ray CT images.

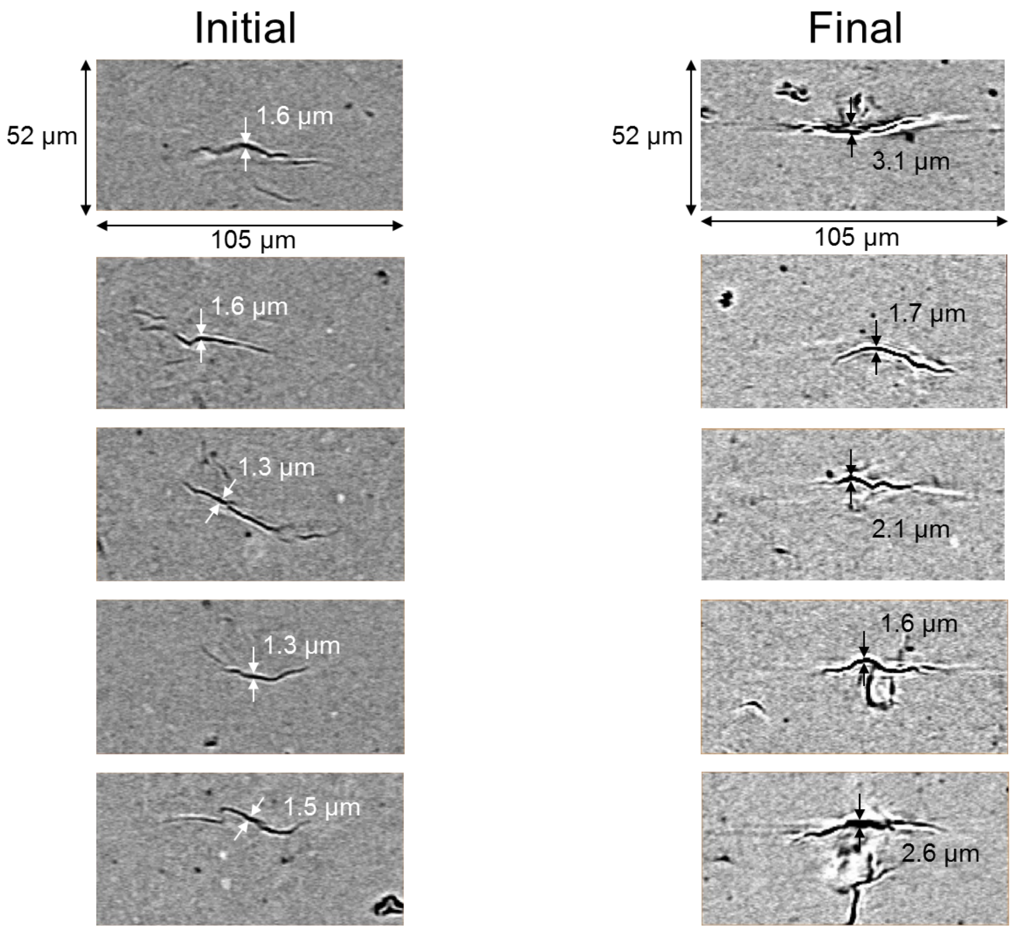


**Supplementary Figure S8.** COD of top 5 largest type III defects at the initial ($\rho$ = 65%) and final ($\rho$ = 98%) stages of sintering.

| Diameter (μm) | Initial stage | Final stage |
|---|---|---|
| 20~25 | 41 | 59 |
| 25~30 | 20 | 44 |
| 30~35 | 20 | 28 |
| 35~40 | 6 | 8 |
| 40~45 | 3 | 6 |
| 45~50 | 4 | 5 |
| 50~55 | 0 | 6 |
| 55~60 | 0 | 1 |
| 60~65 | 0 | 0 |
| 65~70 | 0 | 0 |
| 70~75 | 0 | 1 |
| 75~ | 0 | 0 |

**Supplementary Table 1.** Type III defect population in alumina at the initial ($\rho$ = 65%) and final ($\rho$ = 98%) stage of sintering in the observed volume of 420×420×1050 $\mu m^3$.

| RANK | Initial stage (μm) | Final stage (μm) |
|---|---|---|
| 1 | 49.2 | 72.0 |
| 2 | 48.5 | 57.5 |
| 3 | 47.1 | 54.8 |
| 4 | 45.8 | 54.7 |
| 5 | 44.5 | 54.4 |
| 6 | 41.2 | 52.1 |
| 7 | 41.0 | 52.1 |
| 8 | 38.9 | 50.4 |
| 9 | 38.1 | 49.6 |
| 10 | 37.4 | 47.0 |

**Supplementary Table 2.** Diameter of top 10 largest Type III defects in alumina at the initial (ρ = 65%) and final ($\rho$ = 98%) stage of sintering in the observed volume of 420 × 420 × 1050 $\mu m^3$.

| Diameter (μm) | Initial stage | Final stage |
|---|---|---|
| 4~5 | 111 | 92 |
| 5~6 | 58 | 68 |
| 6~7 | 30 | 31 |
| 7~8 | 8 | 13 |
| 8~10 | 6 | 10 |
| 10~12 | 0 | 3 |
| 12~14 | 1 | 2 |
| 14~16 | 0 | 0 |
| 16~18 | 0 | 1 |
| 18~20 | 0 | 3 |

**Supplementary Table 3.** Type I defect population in alumina at the initial ($\rho$ = 65%) and final ($\rho$ = 98%) stage of sintering in the observed volume of 210×210×210 μm$^3$.
